# Paroxysmal Supraventricular Tachycardia

**DOI:** 10.7759/cureus.4883

**Published:** 2019-06-11

**Authors:** Casey Arnold, Carmen J Martinez Martinez

**Affiliations:** 1 Emergency Medicine, Advent Health Florida Hospital, Orlando, USA

**Keywords:** supraventricular tachycardia, troponin, discharge, modified vagal maneuver

## Abstract

Supraventricular tachycardia is a common emergency department (ED) pathology that frequently leads to hospital admission, but this may not be necessary in all cases. Here, we present a supraventricular tachycardia patient who was discharged from the ED after vagal maneuvers. This case demonstrates evidence that judicious emergency physicians can discharge supraventricular tachycardia patients home safely and gives impetus for a data-driven protocol for discharging these patients.

## Introduction

Supraventricular tachycardia is an arrhythmia frequently encountered by emergency physicians and accounts for over 50,000 cases in emergency departments (EDs) in the United States per year [1]. Multiple approaches have been described for the management of patients presenting with supraventricular tachycardia, but most patients are cardioverted with intravenous (IV) drugs or electricity and admitted to the hospital. We present the case of a 66-year-old male patient with supraventricular tachycardia who was discharged from the emergency department following vagal maneuver cardioversion and a short, two-hour observation period in the emergency department. This case illustrates the possibility of discharging patients with supraventricular tachycardia from the emergency department based on risk factors and suggests the possibility of researching a data-driven protocol for discharging these patients similar to those available for other common presentations.

## Case presentation

**Figure 1:**
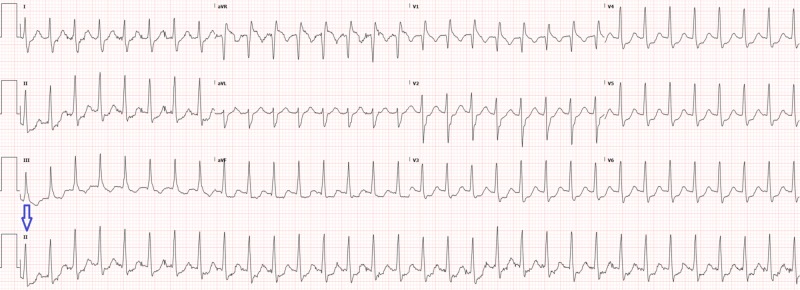
Electrocardiogram on initial presentation with rhythm strip (arrow) showing supraventricular tachycardia

A 66-year-old male with a past medical history of hyperlipidemia, hypertension, and frequent episodes of supraventricular tachycardia presented via ambulance with a chief complaint of tachycardia. The patient was eating lunch at work that day when he started to experience palpitations. Typically, palpitations resolved by taking deep breaths and trying to relax, but, today, this did not resolve his symptoms, so he called an ambulance who did not attempt cardioversion before transport to the ED due to timing. On arrival to the emergency department, the patient’s initial vital signs showed a heart rate in the 180s, but, otherwise, the vital signs were within normal limits. History revealed that the patient complained of dyspnea and anxiety but, otherwise, his review of systems was negative for chest pain, dizziness, cough, and fever. His physical exam was notable for anxiety and regular rhythm tachycardia on auscultation but was otherwise unremarkable. The patient reported that he had a cardiologist and denied any coronary artery disease history. The initial electrocardiogram is shown in Figure 1.

**Figure 2:**
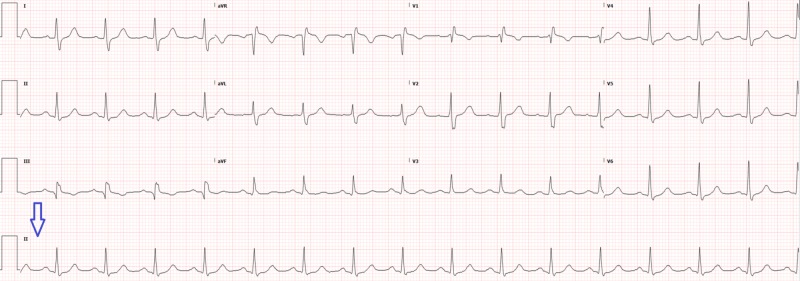
Repeat electrocardiogram two hours later with rhythm strip (arrow) showing normal sinus rhythm

The patient was treated with 1 liter of normal saline and modified vagal maneuvers, as shown in the REVERT trial [2], which converted him to sinus rhythm without medications (Figure 2). The workup performed included hemogram and electrolytes but no cardiac enzymes. The patient was discharged to follow-up with his cardiologist outpatient in two to three days and had no further complications.

## Discussion

Supraventricular tachycardia is a common condition that tends to affect patients, recur at times, and then spontaneously resolve for long periods before affecting patients again. Many times, these patients are admitted to the hospital where they are simply observed. However, here, we present the case of a patient who was discharged home post-cardioversion after several factors were considered. These factors supporting discharge include no chest pain, no history of coronary artery disease, a long history of supraventricular tachycardia (SVT) followed by a cardiologist, with similar symptoms in the past, and an arrhythmia that resolved in the ED without any cardiac medications or electricity.

The main reason the patient would need to be sent to observation is to rule out cardiac ischemia. As previously mentioned, other than his arrhythmia, the patient was asymptomatic, and a quick assessment of his risk factors determined his disposition. When called, his cardiologist confirmed that the patient had a normal stress test six months ago when he had his first episode of supraventricular tachycardia. This made the diagnosis of acute coronary syndrome in this patient lower on the differential. Additionally, this patient’s post-conversion electrocardiogram was normal, without signs of ischemia.

The next test considered was a troponin, but the implications of this test can be far-reaching and misleading if used incorrectly in SVT. Often, in the ED, troponins are ordered in order to rule out ischemia from a focal coronary source, but the increased troponin in supraventricular tachycardia is usually global, demand, or rate-related ischemia, which resolves upon cardioversion [3]. There is a debate on the usefulness of troponins in supraventricular tachycardia patients because increased troponins and even ST-depression on an electrocardiogram are not always accurate markers of acute coronary syndrome [3-4]. Alternatively, other studies show that patients with supraventricular tachycardia and elevated troponins have increased mortality and morbidity rates, but most of these studied patients had other comorbidities such as coronary artery disease, chronic obstructive pulmonary disease, and congestive heart failure [5], which, if present, would make one think about admission for these patients regardless of the troponin. The consensus on troponin testing for SVT is inconclusive, but the evidence seems to suggest that troponins risk stratify high-risk patients but should not be used in patients who are low risk and likely to be discharged unless one is aware of the implications of a mild elevation in this setting [6-7]. In other words, one should not hinge their entire paroxysmal supraventricular tachycardia patient’s disposition on a troponin as one may in other cases. Troponins should be ordered selectively for high-risk supraventricular tachycardia patients and do not need to be ordered in every case. In this case, the history from the patient's cardiologist made the patient lower risk but a personal cardiologist is seldom available for all patients in the ED.

## Conclusions

Currently, there is a push to algorithmizing the practice of emergency medicine, and a one-size-fits-all disposition approach does not work for certain diagnoses like supraventricular tachycardia. The episodic nature of supraventricular tachycardia means that once the patient is cardioverted, there is no further reason to keep the patient in the hospital unless new symptoms occur, indicating a different process. Supraventricular tachycardia usually does not indicate coronary artery disease. Emergency physicians should order troponins only judiciously because ordering them may cause unnecessary admissions and even coronary catheterizations for patients who likely do not have coronary artery disease. Further studies and data collection could create a clinical pathway algorithm for discharging these patients that is similar to those protocols available for other common presentations in the ED.
